# Learning the Way of Health

**Published:** 1924-10

**Authors:** 


### LEARNING THE WAY OF HEALTH.

### HYGIENE FOR SCHOOLS.

If the interest of boys and girls can be excited in the science and art of healthy living, and sustained by a series of talks by (and with) a well-informed teacher, a great step forward will have been taken in the direction of securing the health, and therefore the happiness, of the generations of the future. It is with this object that the Headmasters in Conference resolved that it is desirable—the word "essential" would not have been misplaced—that some systematic teaching on the subject of Hygiene should form part of the instruction in every school. It is in pursuance of the same object that a syllabus—("The Practice of Health." Warren & Son, Ltd., Winchester. 1s., post free.)—has been issued, drawn up by medical experts for the Headmasters' Conference. It is a guide for the teacher as to the arrangement of his subject matters, upon which he must inform himself more fully from other sources. It is a valuable guide, and one to which we all may look with advantage if we would learn something of the simple lessons that it holds between its covers.

### The Quest of Health.

It is recognised as common ground that the practice of health cannot be taught in a set course of lessons :—

Personal and communal hygiene, or more briefly health, should appeal to the boy and girl as a quest, something to be explored rather than a subject in which to receive instruction. Therefore, let the pupil discover the application and advantages of hygiene in his own life, in his school, his home and his workshop. Let him see what true cleanliness means in all its manifold forms, that it constitutes the very foundation of personal and communal health, and that dirt brings disease. Let him discover for himself the relation between physical fitness and output of any kind, whether in games or in work.

### Teaching the Child to Observe.

The poor child will find himself puzzled by the contradiction between the theories of healthy living and the facts of life ; but it will be something accomplished to set him thinking :—

Lead him to see the forms and expressions of hygiene or its absence in the school, the house, the street, the village or the town, the shire, and the country in which he lives, and its far-reaching waves of influence oversea. In a word, excite his interest in the science and art of healthy living, which, with all its common and unattractive details, is, after all, the most vital and human subject of the whole curriculum, for it is concerned not with books or examinations, but with his very life and well-being.

### A Rich Field of Interest.

We doubt whether the interest of children is to be aroused in the Organisation of the Public Health Service (Section II.). The unfortunate teacher may himself become bewildered by the study of the machinery of local government before he gets very far, and decide at any rate to postpone this part of his subject. In regard to Housing and Town Planning, the importance of fresh air and sunlight, and the supply of pure water and pure milk, the disposal of refuse and waste, and the protection of the worker at his work, however, there is a rich field for sustaining the real interest of the child. Moreover, we are glad to see that recognition is made of the value of demonstrations outside the school :—

Wherever possible the practical side of the subject should be directly investigated : excursions may be made to neighbouring water works, arrangements for sewage disposal, &c. ; experimental work may be carried on in the school garden and laboratory ; full use should be made of museums. No stone should be left unturned to make the subject live.

### A Fascinating Study.

The correspondence of functions in animal and plant life and the relations of both to health and disease can be presented in a most fascinating manner. Explanations may be simply given of some of the common ailments and why they develop, of the functioning of the human body, the value of physical training and rest. We hope that we shall not be understood as advocating a smattering of anatomy, and a smattering of physiology, and the rest of it. It is appalling to think of the lost opportunities in schools of presenting attractively the elements of the practice of health, while time is devoted to imparting a little knowledge here and a little there which, whether it be a dangerous thing or not, is at least soon forgotten. The part played by the consumption of suitable food and drink, in keeping the body fit, the value of cleanliness and the disasters for which uncleanliness is responsible, the lessons of heredity, and other subjects are well and briefly handled in the syllabus.

### A World of Romance.

Finally, the history of the securing and maintaining of the public health can be presented to the child as a veritable romance :—

Let the boy and girl hear of the wonderful work by which modern science has built up our knowledge and practice of hygiene—how lice spread typhus and trench fever, how flies are concerned in the spread of typhoid fever, how mosquitoes carry malaria and rats the plague ; some of the outstanding facts of the unseen world of friends and foes wrested from nature by Pasteur and applied by Lord Lister in the saving of millions of men's lives ; the application of hygiene in the war zone by which our soldiers were kept fed and warm, fit and well, in spite of their deprivations ; the steps which have been taken in England with such remarkable success to save the frail lives of new-born infants ; the extraordinary growth in child welfare from the dark and wretched times of the Industrial Revolution down to the present day ; the care which is bestowed on the industrial worker to provide for the maintenance of his health and efficiency in the workshop. There is no subject in the school curriculum which has a more romantic story behind it than the triumphs of the progress of hygiene in recent years, and the story has the advantages of being always new and expanding, It can be told in the simplest language, and it is woven into the geography and history of the world.

---

**Hospital's Lack of Breathing Space.**

Sickness has been experienced by the nursing staff at the Enfield War Memorial Hospital, and it seems possible that their health may be affected by the absence of any grounds for games, rest or exercise. When their work is done they have nowhere to go, except to the town, and the time occupied going and returning exhausts their leisure. The gardens of friends of the hospital have been thrown open to the nurses, but nothing can replace a breathing-ground attached to the hospital itself. Despite its name, the hospital is not a new one, and this may explain its curious want of space.

